# Associations of circulating dimethylarginines with the metabolic syndrome in the Framingham Offspring study

**DOI:** 10.1371/journal.pone.0254577

**Published:** 2021-09-07

**Authors:** Ibrahim Musa Yola, Carlee Moser, Meredith S. Duncan, Edzard Schwedhelm, Dorothee Atzler, Renke Maas, Juliane Hannemann, Rainer H. Böger, Ramachandran S. Vasan, Vanessa Xanthakis

**Affiliations:** 1 Section of Preventive Medicine and Epidemiology, Department of Medicine, Boston University School of Medicine, Boston, MA, United States of America; 2 Center for Biostatistics in AIDS Research, Harvard T.H. Chan School of Public Health, Boston, MA, United States of America; 3 Department of Biostatistics, University of Kentucky, Lexington, KY, United States of America; 4 Department of Clinical Pharmacology and Toxicology, University Medical Center Hamburg-Eppendorf, Hamburg, Germany; 5 German Center for Cardiovascular Research (DZHK), Partner Site Hamburg/Kiel/Lübeck, Hamburg, Germany; 6 Institute for Cardiovascular Prevention, Ludwig-Maximilians-Universität, Munich, Germany; 7 Walther Straub Institute of Pharmacology and Toxicology, Ludwig-Maximilians-Universität, Munich, Germany; 8 DZHK (Deutsches Zentrum für Herz-Kreislauf-Forschung e.V.), Partner Site Munich Heart Alliance, Munich, Germany; 9 Institute of Experimental and Clinical Pharmacology and Toxicology, Friedrich-Alexander-University of Erlangen-Nürnberg, Erlangen, Germany; 10 Framingham Heart Study, Framingham, MA, United States of America; 11 Department of Epidemiology, Boston University School of Public Health, Boston, MA, United States of America; 12 Boston University Center for Computing and Data Sciences, Boston, MA, United States of America; 13 Department of Biostatistics, Boston University School of Public Health, Boston, MA, United States of America; Technische Universität Dresden, GERMANY

## Abstract

**Background:**

Circulating levels of the endogenous inhibitor of nitric oxide synthase, asymmetric dimethylarginine (ADMA), are positively associated with the prevalence of metabolic syndrome (MetS) in cross-sectional investigations. It is unclear if circulating ADMA and other methylarginines are associated with incident MetS prospectively.

**Methods:**

We related circulating ADMA, symmetric dimethylarginine (SDMA), L-arginine (ARG) concentrations (measured with a validated tandem mass spectrometry assay) and the ARG/ADMA ratio to MetS and its components in 2914 (cross-sectional analysis, logistic regression; mean age 58 years, 55% women) and 1656 (prospective analysis, Cox regression; mean age 56 years, 59% women) individuals from the Framingham Offspring Study who attended a routine examination.

**Results:**

Adjusting for age, sex, smoking, and eGFR, we observed significant associations of ADMA (direct) and ARG/ADMA (inverse) with odds of MetS (N = 1461 prevalent cases; Odds Ratio [OR] per SD increment 1.13, 95%CI 1.04–1.22; and 0.89, 95%CI 0.82–0.97 for ADMA and ARG/ADMA, respectively). Upon further adjustment for waist circumference, systolic and diastolic blood pressure, glucose, high-density lipoprotein cholesterol, and triglycerides, we observed a positive relation between SDMA and MetS (OR per SD increment 1.15, 95% CI 1.01–1.30) but the other associations were rendered statistically non-significant. We did not observe statistically significant associations between any of the methylarginines and the risk of new-onset MetS (752 incident events) over a median follow-up of 11 years.

**Conclusion:**

It is unclear whether dimethylarginines play an important role in the incidence of cardiometabolic risk in the community, notwithstanding cross-sectional associations. Further studies of larger samples are needed to replicate our findings.

## Introduction

Insulin resistance (IR) is a key component of the metabolic syndrome (MetS), which is characterized by abdominal obesity, impairment of fasting glucose, dyslipidemia, and hypertension [1, 2]. Individuals with MetS are at increased risk of developing type 2 diabetes and cardiovascular disease [3–6], presumably because these people have IR and a higher burden of subclinical atherosclerosis [7]. It is well established that impaired endothelial nitric oxide (NO) production, often a mediator of endothelial dysfunction, is an early step during the development of atherosclerosis and vascular disease [8, 9]. In this context, the endogenous inhibitor of nitric oxide synthase (NOS), asymmetric dimethylarginine (ADMA), has emerged as an independent predictor for vascular disease and mortality [10]. Endothelial dysfunction and ADMA have also been reported to be associated with IR in hypertensive patients [11, 12], and each of the individual components of the MetS has been associated with impaired endothelial function [5, 6, 13]. Consistent with these observations, mice with a genetic disruption of endothelial NOS display hyperlipidemia, hypertension, and IR, whereas mice overexpressing human dimethylarginine dimethylaminohydrolase 1 (DDAH1), the enzyme mainly responsible for degrading ADMA, show increased insulin sensitivity [14, 15]. Therefore, we hypothesized that ADMA-mediated NOS inhibition might be involved in the pathogenesis of cardiometabolic risk. However, although individuals with MetS have higher circulating ADMA levels compared to individuals without MetS in some cross-sectional studies [16–18], to date, no prior investigation has examined the association between ADMA and the incidence of MetS prospectively. Furthermore, very little is known about the association of symmetrical dimethylarginine (SDMA) with cardiometabolic risk because SDMA (which does not directly inhibit NOS) was thought to be biologically inert. Recently, studies have shown SDMA exerts its biological effects by inhibiting cationic amino acid transport into cells leading to limited cellular L-arginine (ARG) uptake [19–21]. Nonetheless, it was shown that SDMA is inversely related to IR in healthy individuals, and with glycemic control in patients with diabetes [22–26]. In order to further elucidate the relations between dimethylarginines and the development of metabolic disease, we investigated the associations of circulating ADMA, SDMA, ARG, and the ARG/ADMA ratio with prevalent and incident MetS in the large, community-based Framingham Offspring Study sample. We hypothesized that higher and lower plasma concentrations of ADMA and ARG, respectively, are associated with higher odds of MetS cross-sectionally and with a higher risk of MetS prospectively.

## Methods

### Study sample

The design and enrollment criteria of the Framingham Offspring Study have been described in detail elsewhere [27]. The present investigation evaluated two samples of the Offspring cohort obtained from attendees at their sixth quadrennial examination cycle (1995–1998): one sample for the cross-sectional analysis of dimethylarginines with prevalent MetS, and another sample for the prospective relations of dimethylarginines and incident MetS.

**Fig 1:**
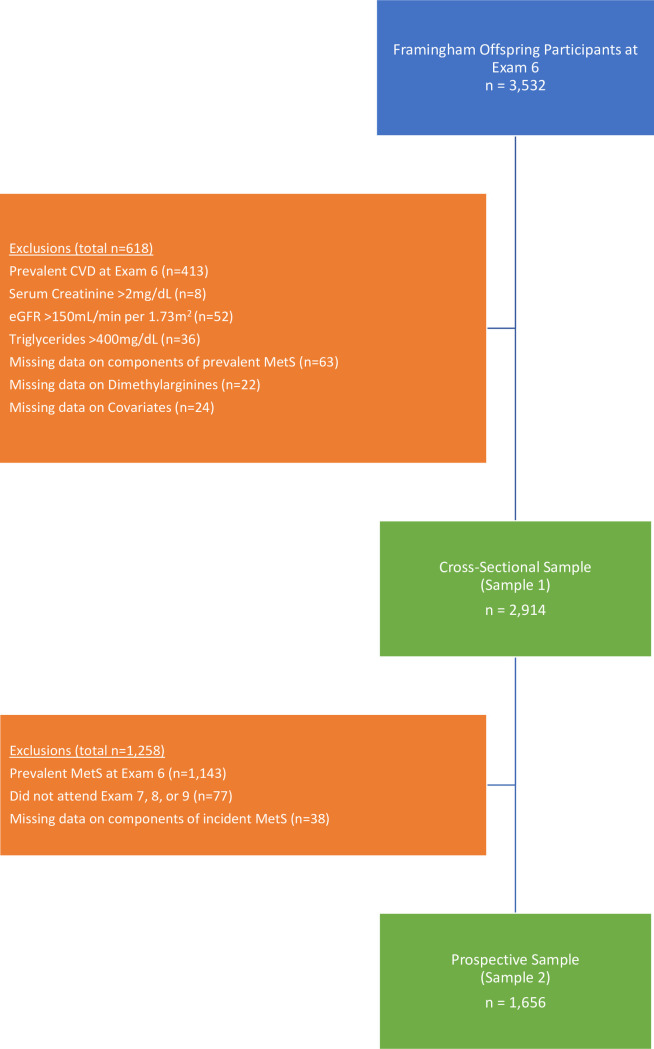
Sample exclusion diagram.

For the cross-sectional analysis, 3532 participants were eligible and 618 individuals were excluded for the following reasons: prevalent cardiovascular disease (coronary heart disease, cerebrovascular disease, intermittent claudication or congestive heart failure; n = 413), serum creatinine >2mg/dL (n = 8), eGFR >150mL/min per 1.73 m^2^ (n = 52), triglycerides > 400 mg/dL (n = 36), missing data on components of MetS (n = 63), missing data on dimethylarginines (n = 22), missing data on covariates (n = 24), resulting in a sample size of 2914 participants (Sample 1) for the cross-sectional analyses (**Fig 1**).

Sample 2, which was used for prospective analyses, was nested within Sample 1. From the 2914 individuals in Sample 1, we additionally excluded participants with prevalent MetS at exam 6 (n = 1143), those who did not attend at least one of the subsequent examinations 7 (1998–2001), 8 (2005–2008) or 9 (2011–2014) (n = 77), and those who were missing components of MetS upon follow-up (n = 38). After exclusions, the final sample consisted of 1656 participants for the prospective analysis (*Sample 2*; **Fig 1**). The study protocol was approved by the Institutional Review Board at Boston University Medical Center and all participants provided written informed consent.

### Assessment of clinical variables and biomarkers of interest

Participants underwent standardized examinations at the Framingham Heart Study research center during which a medical history, a targeted physical examination, anthropometric measurements, and laboratory assessment of cardiovascular risk factors were conducted. Fasting blood samples were obtained from the participants after five to ten minutes rest in a supine position. Plasma samples from the sixth examination cycle, which had been stored without freeze-thaw cycles at -80°C, were used for the measurement of dimethylarginines (ADMA, SDMA) and ARG by liquid chromatography-tandem mass spectrometry (LC-MS/MS) using a stable isotope dilution assay as previously described [28–30].

### Assessment of metabolic syndrome

Prevalent and incident MetS were defined as binary variables according to the modified definition of the National Cholesterol Education Program Adult Treatment Panel guidelines [1]. Participants were classified as having MetS if ≥3 of the following criteria were met: abdominal obesity (waist circumference in men ≥102 cm, in women ≥89 cm), elevated blood pressure (systolic ≥130 mmHg or diastolic ≥85 mmHg) or use of antihypertensive medication; elevated fasting glucose (≥100 mg/dl) or use of anti-hyperglycemic medication; elevated triglycerides (≥150 mg/dl) or use of lipid-lowering treatment; or low HDL cholesterol (men <40 mg/dl, women <50 mg/dl).

### Statistical analysis

Using sample 1, we examined the cross-sectional associations of ADMA, SDMA, ARG, and the ARG/ADMA ratio (independent variables, separate model for each) with the presence of MetS (dependent variable) using two multivariable logistic regression models. The first model was adjusted for age, sex, smoking, and eGFR. The second model additionally included waist circumference, systolic and diastolic blood pressure, fasting glucose, high-density lipoprotein cholesterol, and triglycerides. To evaluate potential nonlinear associations between each of the biomarkers and prevalence of the MetS, we created restricted cubic spline plots with 3 knots at the 10^th^, 50^th^, and 90^th^ percentiles [31].

Next, we evaluated the associations of ADMA, SDMA, ARG, and ARG/ADMA (independent variables, separate model for each) with the incidence of new-onset MetS (absence of MetS at examination cycle 6 and presence of MetS at any of examination cycles 7, 8, or 9) using Cox regression models with discrete time intervals, with examination cycle 6 serving as the baseline, adjusting for the same covariates listed above. We created restricted cubic splines to evaluate potential nonlinear associations between each biomarker and incident MetS. Statistical significance was assessed based on a Bonferroni-adjusted two-sided p-value of <0.0125 (0.05 divided by 4 [= number of methylarginine biomarkers evaluated]). The SAS Software version 9.4 (Cary, NC) was used for all analyses. The authors had full access to the data and take responsibility for its integrity. All authors have read and agree to the manuscript as written.

## Results

### Baseline characteristics

The baseline characteristics of the cross-sectional and prospective study samples (samples 1 and 2, respectively) are displayed in **Table 1**. More than half of the participants of the cross-sectional sample displayed abdominal obesity or elevated blood pressure.

**Table 1:** Characteristics of study participants at the baseline examination for the cross-sectional and prospective analyses.

	Cross-sectional sample		Prospective sample	
	*Men (n = 1310)*	*Women (n = 1604)*	*Men (n = 683)*	*Women (n = 973)*
***Clinical characteristics***				
Age, years	58 ± 10	58 ± 10	56 ± 10	56 ± 9
Smoking, N (%)	185 (14)	237 (15)	97 (14)	141 (15)
Hypertension, N (%)	534 (41)	565 (35)	167 (24)	183 (19)
Diabetes, N (%)	115 (9)	100 (6)	17 (2)	5 (0.5)
Metabolic Syndrome, N (%)	568 (43)	575 (36)	N/A	N/A
Abdominal obesity, N (%)	607 (46)	961 (60)	180 (26)	401 (41)
Elevated BP, N (%)	766 (58)	768 (48)	268 (39)	277 (28)
Elevated triglycerides, N (%)	475 (36)	515 (32)	89 (13)	113 (12)
Low HDL cholesterol, N (%)	493 (38)	483 (30)	112 (16)	133 (14)
Elevated fasting glucose, N (%)	653 (50)	493 (31)	212 (31)	96 (10)
***Laboratory Values***				
BMI, kg/m^2^	28.5 ± 4.4	27.2 ± 5.6	27.0 ± 3.7	25.2 ± 4.2
Waist circumference, cm	101 ± 11	94 ± 15	97 ± 9	88 ± 12
SBP, mmHg	130 ± 17	126 ± 20	124.4 ± 16.0	120 ± 17
DBP, mmHg	78 ± 9	74 ± 9	75.9 ± 8.6	72 ± 9
Fasting glucose, mg/dL	105 ± 24	99 ± 23	98.6 ± 17.3	91 ± 9
Triglycerides, mg/dL	131 ± 68	125 ± 64	104 ± 48	100 ± 48
Total cholesterol, mg/dL	200 ± 35	211 ± 38	200 ± 34	207 ± 38
HDL cholesterol, mg/dL	45 ± 12	59 ± 16	49 ± 12	64 ± 15
eGFR, mL/min	87 ± 17	85 ± 19	87 ± 16	87 ± 18
ADMA, μmol/L	0.55 ± 0.12	0.54 ± 0.13	0.54 ± 0.12	0.53 ± 0.13
SDMA, μmol/L	0.40 ± 0.10	0.39 ± 0.09	0.40 ± 0.09	0.38 ± 0.09
L-arginine, μmol/L	79.8 ± 21.1	77.9 ± 20.4	79.5 ± 20.6	78.4 ± 21.2
Arg/ADMA	150.6 ± 45.3	149.2 ± 43.8	152.4 ± 44.7	152.9 ± 44.0

Data are displayed as means ± standard deviation or frequency and percent (parentheses). Hypertension is defined as SBP/DBP of ≥140/90 or use of anti-hypertension medication. Abdominal obesity is defined among men as a waist circumference ≥102 cm, and among women as a waist circumference ≥89 cm. Elevated BP is defined as SBP/DBP ≥ 130/85 or use of anti-hypertension medication. Elevated triglycerides are defined as ≥150 mg/dL or use of lipid-lowering medication. Low HDL is defined as <40mg/dL for men and <50mg/dL for women. Elevated fasting glucose is defined as fasting glucose ≥100mg/dL or use of anti-hyperglycemic medication.

### Cross-sectional association of ADMA, SDMA, ARG, and ARG/ADMA with prevalent metabolic syndrome

Higher ADMA and lower ARG/ADMA concentrations were associated with higher odds of prevalent MetS, adjusting for age, sex, smoking, and eGFR, but further adjustment for waist circumference, systolic and diastolic blood pressure, glucose, high-density lipoprotein (HDL) cholesterol, and triglycerides rendered the associations statistically non-significant. SDMA was not associated with MetS when adjusting for age, sex, smoking, and eGFR, but further adjustment for the covariates listed above rendered the association statistically significant (**Table 2**). Examination of restricted cubic spline plots did not show significant non-linear associations (**S1 Fig**).

**Table 2:** Association of biomarkers with *prevalent* metabolic syndrome.

	Model 1		Model 2	
*Biomarker*	*OR (95% CI)*	*p-value*	*OR (95% CI)*	*p-value*
ADMA	1.13 (1.04, 1.22)	0.002	1.08 (0.96, 1.22)	0.19
SDMA	0.94 (0.87, 1.02)	0.16	1.15 (1.01, 1.30)	0.032
L-Arginine	0.97 (0.90, 1.05)	0.45	1.05 (0.94, 1.17)	0.39
ARG/ADMA	0.89 (0.82, 0.97)	0.004	0.99 (0.89, 1.11)	0.92

Data are displayed as odds ratios (95% confidence intervals) per 1 standard deviation increase in the respective biomarker.

Model 1 is adjusted for age, sex, smoking, and eGFR.

Model 2 includes the adjustment variables in Model 1 plus waist circumference, SBP, DBP, glucose, HDL cholesterol, and triglycerides.

### Prospective association of ADMA, SDMA, ARG, and ARG/ADMA with incident metabolic syndrome

During a median follow-up of 11 years, 752 individuals developed new-onset MetS (**Table 3**). We did not observe a statistically significant association between any of the methylarginine biomarkers and risk of developing new-onset MetS. As with cross-sectional analyses, examination of restricted cubic spline plots did not show significant non-linear associations between biomarkers and incident MetS (**S2 Fig**).

**Table 3:** Association of biomarkers with *incident* metabolic syndrome.

	Model 1		Model 2	
*Biomarker*	*HR (95% CI)*	*p-value*	*HR (95% CI)*	*p-value*
ADMA	1.02 (0.94, 1.11)	0.61	1.02 (0.94, 1.12)	0.63
SDMA	0.94 (0.86, 1.02)	0.14	1.04 (0.95, 1.14)	0.37
L-Arginine	1.01 (0.93, 1.10)	0.80	1.02 (0.94, 1.11)	0.63
ARG/ADMA	1.00 (0.92, 1.08)	0.94	1.02 (0.93, 1.10)	0.73

Data are displayed as hazard ratios (95% confidence intervals) per 1 standard deviation increase in the respective biomarker.

Model 1 is adjusted for age, sex, smoking, and eGFR.

Model 2 includes the adjustment variables in Model 1 plus waist circumference, SBP, DBP, glucose, HDL cholesterol, and triglycerides.

## Discussion

### Principal findings

Cross-sectionally, higher ADMA and lower ARG/ADMA were associated with higher odds of prevalent MetS adjusting for age, sex, smoking, and eGFR, but further adjustment for additional covariates rendered these associations statistically non-significant. Of note, SDMA was not associated with odds of MetS in minimally-adjusted models (adjusting for age, sex, smoking, and eGFR), but the association became significant in fully-adjusted models.

### Experimental evidence for NOS-inhibition in metabolic disease

Experimental evidence connects impairment of endothelial NO production with metabolic disturbances. Apart from the associations of the genetic models of the eNOS knockout mice and the DDAH1 transgenic mice with insulin sensitivity noted earlier [14, 15], it has been shown that infusion of the NOS-inhibitor N-monomethyl L-arginine (L-NMMA) in rats induced hypertension and insulin-resistance [32]. Moreover, infusion of ADMA into C57BL/6 and apolipoproteinE (ApoE) knockout mice increased plasma triglycerides [33]. Furthermore, there are additional experimental data supporting a causal relation between NOS inhibition and IR. In an experimental setting it was observed that insulin-mediated glucose uptake is closely connected to insulin-mediated, NOS-dependent vasodilation and microvascular recruitment, which in turn are attenuated by NOS-inhibition [34–36]. Moreover, a non-obese IR rat model fed with fructose showed an elevation of circulating and aortic ADMA concentrations, as well as reduced DDAH aortic content and increased aortic arginase activity in IR. Likewise, ARG supplementation and arginase inhibition improve endothelial vasodilatation in IR rats providing further functional corroboration [2].

### Comparison to the literature

#### ADMA and MetS

Several cross-sectional investigations have analyzed ADMA plasma levels in people with MetS while others evaluated the associations of ADMA plasma levels with individual components of MetS. Recent prospective studies have related plasma dimethylarginines to the risk of developing MetS but their findings are not consistent [24–26, 37–39]. Several studies reported that plasma ADMA was not significantly higher in people with MetS [19–21, 40], although the literature has not been entirely consistent in this regard [16–18]. Furthermore, plasma ADMA concentrations have also been directly related to measures of IR such as the homeostasis model assessment (HOMA), insulin suppression test or hyperinsulinemic, and euglycemic clamp in non-diabetic individuals, including healthy people as well as obese, elderly and hypertensive individuals [2, 41–43]. Clinical studies also have reported higher ADMA plasma concentrations with higher values of anthropometric measures of excess adiposity such as body mass index (BMI), waist circumference, body fat mass, and body weight in healthy individuals [44, 45]. This relation between ADMA, obesity, and IR is further supported by interventional studies, which have shown that weight loss was associated with a lowering of circulating ADMA levels in obese individuals, which in turn was accompanied by an increase in insulin sensitivity and NO production [46, 47]. Contrary to these findings, our prospective investigation showed no statistically significant association between ADMA and risk of developing new-onset MetS. Furthermore, the attenuation of the cross-sectional association we observed between ADMA and presence of MetS could be due to the fact that we adjusted the model for variables that are components of the MetS.

#### SDMA and MetS

SDMA has been less well investigated, but there is increased focus with regards to its relation with cardiometabolic diseases. SDMA was inversely associated with the HOMA index in a sample of healthy white individuals [22]. In another report evaluating patients with type 2 diabetes, plasma SDMA levels were inversely associated with glycemic control [23]. Marliss and colleagues reported that, similar to ADMA, plasma SDMA levels were positively related with IR and triglycerides, and inversely related with HDL cholesterol [42]. In our investigation, we observed a direct association of SDMA with prevalent but not incident MetS, which is consistent with some prior reports [19–21, 25, 26]. Moreover, SDMA was related to MetS cross-sectionally in a fully-adjusted model but not a minimally adjusted model, perhaps suggesting the presence of reverse confounding by HDL [48, 49].

The mechanisms by which SDMA exerts its biological effects is by inhibiting cationic amino acid transport into cells leading to limited cellular l-arginine uptake [19–21]. Additionally, there are some experimental studies connecting SDMA to inflammation and oxidative stress [50, 51], but additional prospective studies are needed to elucidate how SDMA may be directly related to the development of metabolic disturbances.

### Strengths and limitations

The strengths of our investigation include its design with both cross-sectional and prospective components, and the large community-based and well-phenotyped sample. Furthermore, the routine assessment of clinical and laboratory data, including validated assays for dimethylarginines, enabled us to perform multivariable analysis adjusting for relevant covariates. However, our sample consisted predominantly of white, middle-aged individuals, so our results may not be generalizable to other races and ethnicities. Moreover, it is possible that dimethylarginine concentrations fluctuated between exam 6 and the time when assessment of incident metabolic syndrome was performed; however, dimethylarginines were only measured at examination cycle 6, therefore we are not able to account for such fluctuations.

## Conclusions

In our investigation of a large prospective, community-based sample, it is not clear whether dimethylarginines play an important role in the pathogenesis of cardiometabolic risk in the community.

## Supporting information

S1 Fig Association between biomarkers and prevalent metabolic syndrome: Restricted cubic spline plots. (DOCX)

S2 Fig Association between biomarkers and incident metabolic syndrome: Restricted cubic spline plots. (DOCX)
