# Supplementary material for: Associations of circulating dimethylarginines with the metabolic syndrome in the Framingham Offspring study
Source: PLoS One. 2021 Sep 7;16(9):e0254577. doi: 10.1371/journal.pone.0254577 (PMC8423279; doi:10.1371/journal.pone.0254577)

**S2 Fig.** Association between Biomarkers and Incident Metabolic Syndrome: Restricted Cubic Spline Plots


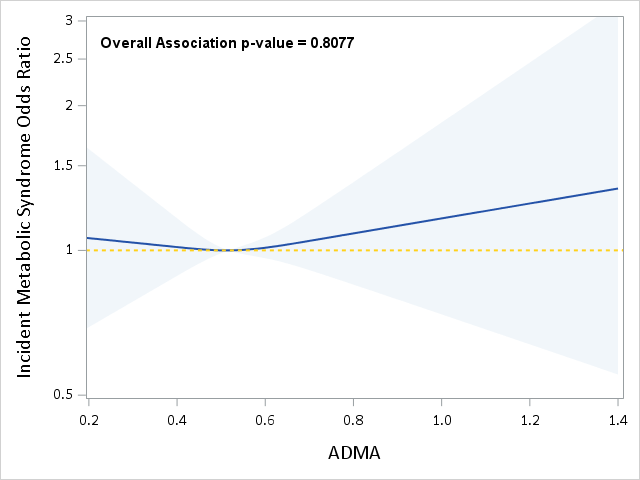

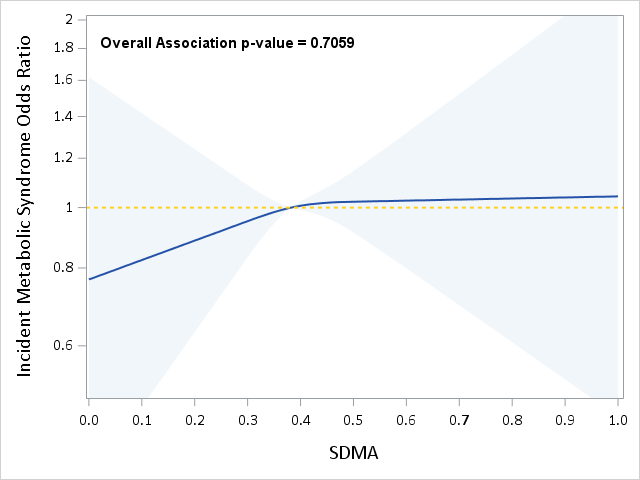


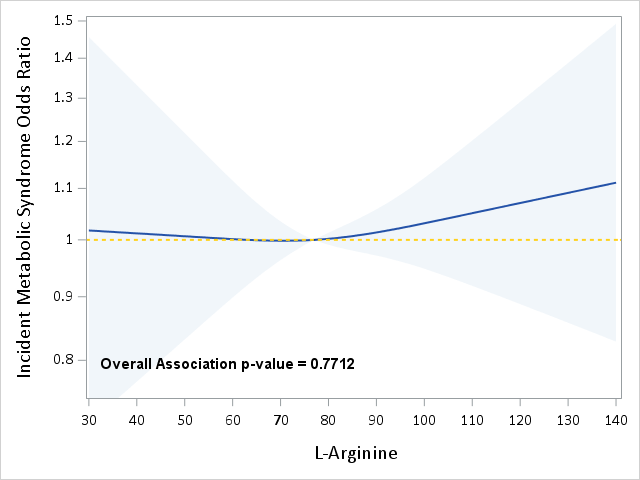

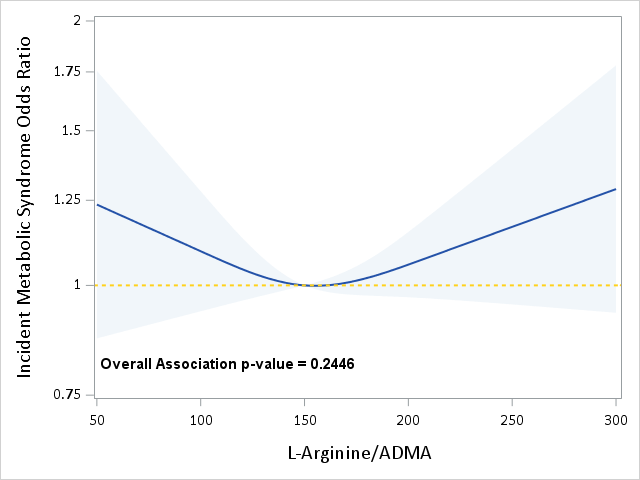

Supplement: S2 Fig — (DOCX) [file pone.0254577.s002.docx]
